# Internet Addiction and Emotional and Behavioral Maladjustment in Mainland Chinese Adolescents: Cross-Lagged Panel Analyses

**DOI:** 10.3389/fpsyg.2021.781036

**Published:** 2021-11-04

**Authors:** Xiaoqin Zhu, Daniel T. L. Shek, Carman K. M. Chu

**Affiliations:** Department of Applied Social Sciences, The Hong Kong Polytechnic University, Kowloon, Hong Kong SAR, China

**Keywords:** internet addiction, depression, delinquency, longitudinal study, junior secondary school students

## Abstract

Adolescence is a developmental stage when adolescents are vulnerable to addictive behaviors, such as Internet addiction (IA), which refers to pathological use of the Internet. Although there are views proposing that the links between IA and adolescent problem behavior may be bidirectional in nature, few studies have examined the reciprocal relationships between IA and other maladjustment indicators, and even fewer studies have simultaneously employed both emotional and behavioral maladjustment indicators in a single study. To address the above research gaps, the present study investigated how IA is associated with both depression and delinquency among Chinese adolescents. Two waves of data were collected at two consecutive years, respectively, with 1year apart, from 3,010 students (Mean age=13.16, SD=0.81; 57.48% boys) in four junior high schools in mainland China. These students completed the same questionnaire containing measures of IA, depression, and delinquency at each wave. The proposed cross-lagged panel model fitted the data very well, and there were significant positive reciprocal effects between IA and depression as well as delinquency after controlling for background socio-demographic factors. Gender differences were also observed in multi-group comparisons. Specifically, IA showed a stronger longitudinal impact on delinquency among boys than among girls. While depression significantly predicted IA in 1year among boys, such a prediction was not significant among girls. These findings delineate the bidirectionality of the associations between IA and emotional and behavioral maladjustment indexed by depression and delinquency, respectively. The findings also suggest that researchers and practitioners have to take gender differences as well as different developmental indicators in understanding the bidirectional influences between IA and adolescent behavioral and emotional development.

## Introduction

The Internet has become an essential part of people’s daily life, including children and adolescents. For example, 25.4% of the children aged 10–14 in Hong Kong were found to spend 20–50h per week using the Internet in 2007; the figure increased to 42.0% in 2017 (Chung et al., 2019). With the fast-growing number of Internet users and increasing time online, the problem of Internet addiction (IA) and its adverse health consequences have become a major public concern (Caplan, 2003). IA, also known as “problematic Internet use,” “pathological Internet use,” or “compulsive Internet use,” is characterized by having an excessive level of preoccupation or behaviors regarding Internet usage, which would usually result in distress and functional impairments (Weinstein et al., 2014). Some researchers considered IA a type of psychological disorder under the category of impulse-control and/or obsessive–compulsive disorder, as it shows symptoms similar to many substance use disorders, including “excessive use,” “withdrawal phenomena,” “tolerance,” and “negative repercussions” (Young, 1998). However, Pies (2009) argued that there are no well-established physiological measures (e.g., blood pressure) or data for withdrawal or tolerance among individuals with IA. Further, as Internet is merely a medium or platform rather than a substance, the pathological need to use Internet may merely represent a behavioral manifestation of psychopathology or defense mechanisms (Pies, 2009). The enduring debate regarding the definition of IA has been mentioned in many studies, and scholars agree that the diagnostic criterion of IA is still open for discussion (Pies, 2009; Stip et al., 2016; Yu and Shek, 2018; Martins et al., 2020). However, a common consensus is that individuals can display different levels of addicted behavior related to Internet use, which has been commonly operationally conceived as a continuous construct indicated by integrated scores in different assessment tools (Liang et al., 2016; Yu and Shek, 2018; Tian et al., 2021).

Across various age groups, adolescents are most vulnerable to develop IA since they commonly lack effective self-regulation ability to control Internet surfing and they tend to be risk-taking and impulsive (Cerniglia et al., 2017). Indeed, a relatively high and growing prevalence of IA among adolescents has been reported in both East and West societies, with the problems being most prevalent in Asian societies (Chung et al., 2019; Pan et al., 2020). In China, Wu et al. (2016) revealed a 10.4% prevalence rate of IA among 14–24-year-old young people in Anhui province; Xin et al. (2018) reported that the overall IA prevalence among adolescents aged 10–18years in Guangzhou was as high as 26.5%; Xu et al. (2020) found that 32.5 and 19.8% of secondary school students in Macau and mainland China showed IA problems, respectively.

The worrying picture of the IA problem is not limited to its high and growing prevalence, but also related to its comorbidity with a wide range of other developmental issues. Ample findings demonstrated that IA co-exists with multiple adverse developmental outcomes. It was reported that IA and other addictive problems (e.g., gaming or substance addiction) shared common cognitive and behavioral characteristics, such as reward deficiency and impulsivity, changes in brain functions, such as hyperactivity of nucleus accumbens but diminished activity of ventral medial prefrontal cortex (Jorgenson et al., 2016; Cerniglia et al., 2017). In addition, IA was associated with social, emotional, and behavioral maladjustment. For example, In Xu et al.’s (2020) study, students categorized as having IA showed poorer academic performance and relationships with others (e.g., classmates, teachers and family), as well as more severe depressive symptoms and physical health issues. The positive linkage between IA and depression was also reported in many other studies (Kim et al., 2008; Wartberg et al., 2016; Wu et al., 2016). Furthermore, misconduct, such as delinquency characterized by rule-breaking or offenses carrying risks to oneself, others, family, school, and society, has also been found to be associated with IA. For example, Kim et al. (2008) considered delinquent behavior as one factor explaining significant variance in IA among Korean adolescents. Positive associations between IA and delinquent problems were also identified in other places such as China (Guo et al., 2021) and Europe (Evren et al., 2014; Wartberg et al., 2016).

Despite the observation on the association between IA and other maladjustment, the causal effects between these two domains remain inconclusive. On the one hand, the mood enhancement hypothesis (Zillmann, 1991) suggests that the use of Internet depends on the user’s mood. When an individual has developmental problems, such as depression and delinquency, he or she is more likely to experience negative feelings and frustrations in the social world. As a result, the individual would prefer interacting with others online rather than off-line as the former is less threatening (Xin et al., 2018). The Internet -mediated virtual world provides a more comfortable and safer environment where the depressed or delinquent adolescents can “meet” and share feelings with others who may have similar experiences. To some extent, Internet is used to relieve negative feelings and escape real-life problems. In such a context, adolescents with developmental adjustment problems may gradually spend more time online, leading to excessive and compulsive use of Internet. In this line of reasoning, most previous studies considered IA a consequence of other developmental problems (Evren et al., 2014; Lam, 2014; Fayazi and Hasani, 2017).

On the other hand, a growing body of literature has also regarded IA as a predictor of negative development (Lam and Peng, 2010; Shek et al., 2018a; Yu and Zhou, 2021). This line of research usually upholds the social displacement hypothesis (Kraut et al., 1998). This framework suggests that the overuse of Internet would compete one’s time and energy to engage in face-to-face interactions with friends and family, creating neglect of daily routines, impairment of social skills, isolation from social life, which eventually leads to social and emotional problems such as depression and delinquency (El Asam et al., 2019). In line with this notion, IA was found to negatively influence adolescents’ social and emotional skills (Brunborg and Andreas, 2019; Yu and Zhou, 2021). Furthermore, young people’s social comparisons through online platforms (e.g., social media) tend to be unrealistic, resulting in feelings of failure and frustration that would create subsequent emotional and behavioral problems (Brunborg and Andreas, 2019).

Although there are theoretical accounts and empirical evidence suggesting the bidirectional relationships between IA and depression and delinquency, only few longitudinal studies have tested this possibility (particularly depression) and the findings are not consistent. For example, van den Eijnden et al. (2008) found no significant cross-lagged predictions between IA and depression in a 6-month longitudinal study. Gámez-Guadix (2014) only found a reciprocal relationship between depressive symptoms and one specific component of IA (i.e., social problems associated with Internet use) but no such effect was found between depression and other components of IA, such as deficient self-regulation and preference for online interactions. In a more recent study, IA was found to significantly predict adolescents’ hopelessness and low satisfaction but no evidence was found for the reverse effects (Yu and Shek, 2018). Nevertheless, another two recent studies identified significant reciprocal predictions between IA and depression among Chinese adolescents (Lau et al., 2018; Tian et al., 2021). While the bidirectional relationship between IA and depression is equivocal, no research to date has ever investigated the reciprocal effects between IA and delinquency. As depression and delinquency are important indicators of adolescent maladjustment in emotional and behavioral domains, respectively, there is a call for more longitudinal studies in this field to test the reciprocal relationships between IA and both depression and delinquency.

The previously mentioned equivocal findings regarding the IA-depression association may be partially attributable to gender effect, which was not examined in those studies showing inconsistent findings. While some studies observed that adolescent boys reported higher levels of IA but a lower level of depression than did adolescent girls (Evren et al., 2014; Ha and Hwang, 2014; Bağatarhan and Siyez, 2020), other studies identified no gender difference in the prevalence of IA (Yu and Zhou, 2021) or depression (Zhu et al., 2021). Similarly, gender differences in the relationship between IA and depression also remain unclear. Liang et al. (2016) identified a significant prediction of depression on subsequent IA only among boys but not among girls. The authors thus argued that boys may be more likely to turn to Internet for mood regulation, while girls may tend to seek help from others in the surrounding environment when they are not happy. However, in another two studies, girls with depressive symptoms were at a higher risk of developing IA than boys with similar problems (Ha and Hwang, 2014; Bağatarhan and Siyez, 2020). The authors also found that most girls used Internet for social networking, whereas boys used it for entertainment (e.g., playing online games). This observation suggests that when girls are faced with emotional difficulties, they are also likely to express unhappy feelings through the Internet.

As for delinquency, while adolescent boys have been found to typically report more delinquent behavior than do adolescent girls (Kofler et al., 2011; Stockdale and Coyne, 2020; Zhu and Shek, 2021a), gender differences in IA-delinquency association remain unknown. Some scholars contended that compared with girls, boys are more likely to act out stresses, difficulties, or unhappy feelings through deviant and rule-breaking behavior such as delinquency (Kofler et al., 2011). In this sense, boys, rather than girls, are more likely to express negative experiences related to IA through delinquent behavior, leading to a stronger IA-delinquency association among boys than among girls. Unfortunately, no research has ever examined this possibility. In view of the current inconclusive findings and the lack of research, it is necessary to investigate gender differences in the bidirectional relationships between IA and maladjustment in different domains.

In response to the call for addressing the above-mentioned issues, we attempted to investigate the possible reciprocal effects between adolescent IA and their emotional (i.e., depression) and behavioral (i.e., delinquency) maladjustment simultaneously in one single study. To do so, cross-lagged panel modeling (CLPM) analyses were carried out to analyze two waves of longitudinal data. In view of the existing literature showing positive linkages between IA and other problem behaviors (Lam, 2014; Liang et al., 2016), we hypothesized positive reciprocal relationships between IA and both delinquency (Hypothesis 1) and depression (Hypothesis 2). We would also explore gender invariance on the expected reciprocal impacts using multi-group comparisons.

**Figure 1:**
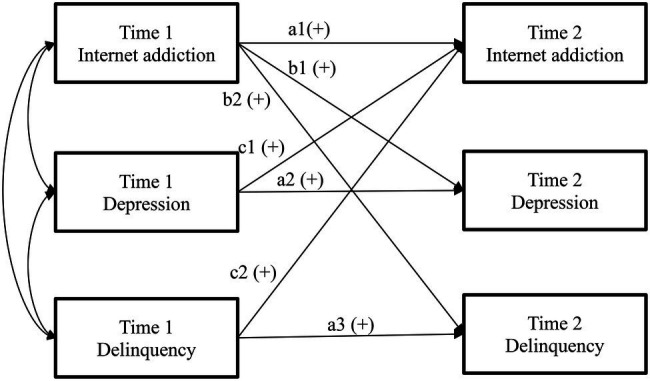
Conceptual models (residuals and covariates are omitted for parsimony). Model 1: No cross-lagged associations (b and c paths are dropped). Model 2: Internet addition effects model (c paths are dropped). Model 3: Depression and Delinquency effects model (b paths are dropped). Model 4: Reciprocal effects model (all paths are included).

Following previous practice (Yu and Shek, 2018; Zhu and Shek, 2021b), we tested four competing models (see Figure 1) to check whether the reciprocal effects model would best fit the data. In Model 1, only autoregressions were modeled (i.e., no predictions effects between IA and other problem behavior). Compared with Model 1, prospective effects of IA on depression and delinquency were further specified in Model 2, while prospective effects from depression and delinquency to IA were added in Model 3. The last model (Model 4) further modeled reciprocal longitudinal relationships between IA and depression and delinquency. Based on our hypotheses, we expected that Model 4 would show the best model fit.

## Materials and Methods

### Participants and Procedures

We tested the above hypotheses among junior secondary school students (i.e., early adolescents) who have to face growing developmental challenges in different domains, such as academic stress, peer pressure, conflicts with parents, difficulties in managing emotions, and so on. It is widely agreed that IA and emotional and behavioral maladjustment (e.g., depression and delinquency) are more common in adolescents than in children, and there is a steady increase in IA, depression, and delinquency over time among some early adolescents (Houghton et al., 2018; Shore et al., 2018; Yu and Shek, 2018; Zhu and Shek, 2021a). Thus, it is theoretically and practically (e.g., early intervention) meaningful to address the research gaps regarding the relationship between IA and depression and delinquency among this group of students.

This study used two waves of data collected from junior secondary school students in four schools in mainland China (Shanwei, Zhaoqing, Jiujiang, and Suzhou). In September 2016 (i.e., Time 1), a total of 3,010 Grade 7 and Grade 8 students (age range=11–15years; Mean age=13.16, SD=0.81; 57.48% adolescent boys) in these schools completed a survey investigating their psychosocial adjustment (e.g., delinquency, depression, and IA). The majority of these respondents (83.26%) reported that they lived in an intact family. Besides, 2,648 students responded to the same survey in 1year (i.e., Time 2), suggesting a total of 362 dropouts and thus an attrition rate of 12.03%. The retained sample (*N*=2,648) and the dropouts (N=362) did not differ in their demographic attributes (age, gender, and family intactness) and baseline IA, delinquency, and depression. Nevertheless, the whole sample (N=3,010) was used for final data analysis. A 1-year interval between the two assessments is convenient in terms of school administrative operations (i.e., not to disturb the school too frequently), and such “short-term” longitudinal study is not uncommon in the research field (Lam, 2014; Yu and Shek, 2018; Stockdale and Coyne, 2020).

This study obtained ethical approval from the “Human Subjects Ethics Subcommittee” at the authors’ institution. Before launching the project, all involved parties, including schools, adolescents, and parents, were well informed about the study objectives, the confidentiality of data collected, voluntary participation, free withdrawal at any time, and anonymous data analyses and dissemination. All parties had given their consent to join the study before data collection.

### Measures

#### Internet Addiction

The Chinese Internet Addiction Test (CIAT) was translated and validated by Shek et al. (2008) based on Kimberly Young’s 10-item questionnaire that included 10 typical symptoms of IA (Suler, 2004). Students responded “Yes” or “No” to the 10 questions based on their experience in the past 1year. The sample questions included “Do you feel preoccupied with the Internet or online services and think about it while off-line?” and “Have you repeatedly made unsuccessful efforts to control, cut back, or stop Internet use?” Participants’ IA was indexed by the number of “yes” answers they provided in the CIAT. Thus, the score has a possible range between 0 and 10, with a higher score indicating a higher level of IA symptoms. In this study, we treated CIAT scores as continuous variables because cutoff scores were not validated in Hong Kong. Further, this scale has been frequently used in previous studies to assess adolescents’ IA level as a continuous variable (Chi et al., 2016; Shek et al., 2018b; Yu and Shek, 2018; Yu and Zhou, 2021). In this study, Cronbach’s αs of the scale (0.78 and 0.80 at Wave 1 and Wave 2, respectively) indicated adequate scale reliability. McDonald’s omegas (ω) also indicated adequate reliability.

#### Depression

The 20-item validated Chinese “Center for Epidemiological Studies-Depression” scale (CES-D) was employed to measure students’ depression (Radloff, 1977; Zhu et al., 2021). Among the 20 items, 16 items assessed negative symptoms (e.g., “I felt lonely”) and the other four were reversely coded positive items (e.g., “I enjoyed life”). Students indicated the frequency they showed each symptom in the previous week using responses from “0″ (“rarely or less than 1day”) to “3″ (“most or all of the time or 5–7days”). Although the scale items assessed depressive symptoms from different dimensions (e.g., somatic complaints and negative affect), an overall score (e.g., average score or total score) is commonly used to indicate the level of depression in literature (Lau et al., 2018; Chi et al., 2019; Przepiorka et al., 2019). In our study, an average score was computed for each participant to indicate the level of depression. Cronbach’s αs of this scale in the present study were 0.87 and 0.89 at two waves, respectively. McDonald’s omegas (ωs) showed similar results.

#### Delinquency

We measured students’ delinquency in terms of how frequently (“0=never”; “6=more than 10 times”) they had engaged in 12 misconducts (e.g., “cheating,” “stealing,” “running away from home,” and “trespassing”) in the past 1year (Shek and Zhu, 2019). Some of the listed delinquent behaviors, such as “stealing” and “damaging others’ properties,” are illegal behavior violating the law. However, some behaviors, such as “having sexual intercourse with others” and “running away from home,” are minor offenses but still considered adolescent problem behavior in mainland China. The average score across the items was used in the present study. Cronbach’s α values of this scale (0.74 and 0.78 in Wave 1 and Wave 2, respectively) suggest adequate scale reliability in the present study. McDonald’s omegas (ωs) also suggested adequate reliability of the scale.

Several control variables were also measured in this study. These included age, gender, and non-intact family. These control variables were commonly used in previous studies (Yu and Shek, 2018; Zhu and Shek, 2021b).

### Data Analysis

Mplus 8.5 was used to analyze cross-lagged panel models with the “full information maximum likelihood estimation” employed to handle missing values. This method makes use of all available information for each participant, and it has been proved to yield unbiased results in analyzing longitudinal data (Muthén and Muthén, 2018). First, the four competing models shown in Figure 1 were tested using path analysis and adequate model fit was decided by following indices and criteria proposed by Kline (2015): “Comparative Fit Index” (CFI>0.95), “Tucker-Lewis Index” (TLI>0.95), “Root Mean Square Error of Approximation” (RMSEA <0.08), and “Standardized Root Mean Square Residual” (SRMR <0.08). As the four models were nested models, chi-square difference tests were used to examine which model best fit the data (Schermelleh-Engel et al., 2003). Second, multi-group (boys vs. girls) analyses were conducted based on the best-fitted model to explore adolescent gender effect. Before the above-mentioned formal analyses, we also performed confirmatory factor analyses (CFA) with invariance tests across gender and over time for each key measure (i.e., one-factor structure for IA and delinquency and three-factor structure for depression). Results showed that the measures were invariant across gender and over time (i.e., changes in CFI and RMSEA were less than 0.01), allowing us to use the scale score (e.g., the average or total score) of each measure for boys and girls at the two waves in the path analyses of the cross-lagged panel models. As scale validation is not the focus of the present study, related findings have been reported elsewhere (Zhu and Shek, 2020b; Zhu et al., 2021).

## Results

As shown in Table 1, IA was positively correlated with depression and delinquency in both cross-sectional and longitudinal levels after Bonferroni correction (*p*=0.05/9=0.006). There were also positive correlations between depression and delinquency among adolescents. These results are in line with our expectations. Besides, three covariates also had significant associations with adolescent IA, depression, and delinquency, except for an insignificant relationship between gender and depression. Specifically, younger adolescents, or girls, or those who live in intact families reported lower levels of IA and delinquency, and younger or those who live in intact families reported lower levels of depression.

**Table 1:** Descriptions of variables and correlations among the variables.

S. No.	Variables	Descriptions			Correlations							
		Mean	SD	*α/ω*	1	2	3	4	5	6	7	8
1.	Age	13.16	0.81		--							
2.	Gender^a^				−0.08^***^	--						
3.	Family intactness^b^				0.02	0.003	--					
4.	T1 IA	2.31	2.36	0.78/0.78	0.09^***^	−0.15^***^	0.09^***^	--				
5.	T1 DP	1.80	0.49	0.87/0.87	0.08^***^	0.03	0.07^***^	0.33^***^	--			
6.	T1 DE	0.45	0.54	0.74/0.75	0.11^***^	−0.15^***^	0.09^***^	0.38^***^	0.27^***^	--		
7.	T2 IA	2.30	2.44	0.80/0.80	0.06^**^	−0.10^***^	0.05^***^	0.46^***^	0.23^***^	0.25^***^	--	
8.	T2 DP	1.79	0.52	0.89/0.89	0.06^**^	0.04	0.05^**^	0.24^***^	0.47^***^	0.23^***^	0.35^***^	--
9.	T2 DE	0.42	0.57	0.78/0.76	0.07^***^	−0.12^***^	0.06^**^	0.27^***^	0.21^***^	0.43^***^	0.32^***^	0.26^***^

a 1=male and 2=female.

b 1=intact; 2=non-intact; T1=Time 1; T2=Time 2.

** *p*<0.01;

*** *p*<0.001. IA, internet addiction; DP, depression; DE, delinquency.

Results of model comparisons of the four competing models are presented in Table 2. All models demonstrated acceptable model fit. However, Model 2 (with IA effects) and Model 3 (with depression and delinquency effects) yielded a better model fit than did Model 1 (only autoregressions), with *∆χ*^2^_(2)_>32, *p*<0.001. As predicted, Model 4 (i.e., reciprocal effects between IA and emotional as well as behavioral problem) better fitted the data than did Model 2 and Model 3, with *∆χ*^2^_(2)_>25, *p*<0.001. Thus, in line with our expectation, the reciprocal impacts model showed best model fit (*χ*^2^_(11)_=56.517, CFI=0.986, TLI=0.959, RMSEA=0.038, SRMR=0.020). Regression coefficients suggested positive reciprocal effects between IA and depression as well as between IA and delinquency (*β* ranged between 0.06 and 0.13, *p*s<0.001; see Table 3, the whole sample). These results support Hypothesis 1 and Hypothesis 2.

**Table 2:** Model fit indexes and comparison for different models of the relationship between internet addiction and depression and delinquency.

Models	χ^2^	*df*	CFI	TLI	RMSEA (90% CI)	SRMR	Model comparison	∆χ^2^	∆*df*
Model 1: No cross-lagged effects	153.132	15	0.958	0.908	0.056 (0.048, 0.064)	0.045			
Model 2: Internet addiction effects	82.042	13	0.979	0.947	0.043 (0.034, 0.052)	0.028	M2 vs. M1	71.09^***^	2
Model 3: Depression and delinquency effects	120.845	13	0.967	0.917	0.053 (0.045, 0.062)	0.037	M3 vs. M1	32.29^***^	2
Model 4: Reciprocal effects	56.517	11	0.986	0.959	0.038 (0.028, 0.048)	0.020	M4 vs. M1	96.62^***^	4
							M4 vs. M2	25.53^***^	2
							M4 vs. M3	64.33^***^	2
Multi-group (boys vs. girls) tests on the reciprocal model									
Model 4a: Unconstrained model	48.050	16	0.990	0.966	0.037 (0.025, 0.049)	0.022			
Model 4b: Constrain internet addiction → depression	48.827	17	0.990	0.969	0.036 (0.024, 0.048)	0.022	M4b vs. M4a	0.77	1
Model 4c: Constrain internet addiction → delinquency	55.730	17	0.988	0.962	0.039 (0.028, 0.051)	0.024	M4c vs. M4a	7.68^**^	1
Model 4d: Constrain depression → internet addiction	54.975	17	0.988	0.963	0.039 (0.028, 0.051)	0.023	M4d vs. M4a	6.93^**^	1
Model 4e: Constrain delinquency → internet addiction	48.156	17	0.990	0.969	0.035 (0.024, 0.047)	0.022	M4e vs. M4a	0.11	1
Model 4f: free internet addiction → delinquency, and depression → internet addiction, constrain other paths (final model)	48.934	18	0.990	0.971	0.034 (0.023, 0.046)	0.022	M4f vs. M4a	0.88	2

** *p*<0.01;

*** *p*<0.001.

**Table 3:** Regression weights of paths in the reciprocal model for the whole sample, boys, and girls.

Predictor	Dependent variable	β		
		Whole sample	Boys	Girls
Time 1 Internet addiction	Time 2 Depression	0.10^***^	0.11^***^	0.09^***^
Time 1 Internet addiction	Time 2 Delinquency	0.13^***^	0.14^***^	0.07^*^
Time 1 Depression	Time 2 Internet addiction	0.06^**^	0.10^***^	0.02
Time 1 Delinquency	Time 2 Internet addiction	0.06^**^	0.06^**^	0.05^**^

* *p*<0.05;

** *p*<0.01;

*** *p*<0.001.

Several multi-group tests by gender on the reciprocal effects model (i.e., Model 4) were performed to explore adolescent gender effect. Results are shown in Table 2. From Model 4b to Model 4c, one cross-lagged path was constrained to be equal between boys and girls in each comparison model. Compared to the unconstrained model (Model 4a) where all cross-lagged paths were freely estimated, Model 4b (an equality constraint on the cross-lagged path from IA to depression) and Model 4e (an equality constraint on the cross-lagged path from delinquency to IA) did not show significant chi-square differences (*Δχ*^2^_(1)_<3.84, *p*>0.05). However, Model 4c (an equality constraint on the cross-lagged path from IA to delinquency) and Model 4d (an equality constraint on the cross-lagged path from IA to delinquency) yielded significant chi-square differences from Model 4a: *Δχ*^2^_(1)_=7.68 and 6.93 (*p*s<0.01). As such, the final model (Model 4f) freely estimated predictive effects from IA to delinquency as well as that from depression to IA while constrained the other two paths to be equal across gender.

Regression coefficients of cross-lagged paths in Model 4f are depicted in Table 3. Although the positive prospective predictions of IA on delinquency were significant for both boys and girls (boys: *β*=0.14, *p*<0.001; girls: *β*=0.07, *p*<0.05), the effect appeared stronger among boys. In addition, the longitudinal effect of depression on IA was significant only among boys (boys: *β*=0.10, *p*<0.001; girls: *β*=0.02, *p*=0.59). These results implied that the IA and emotional and behavioral problems had reciprocal effects on each other, and some effects were similar among boys and girls while some showed significant gender differences.

## Discussion

The present study investigated cross-lagged effects between Internet addiction (IA) and emotional and behavioral problems (depression and delinquency) over 2years among Chinese adolescents. The results reveal that baseline IA measured at Time 1 significantly and positively predicted depression and delinquency in 1year (i.e., at Time 2). Meanwhile, higher levels of depression and delinquency at Time 1 also significantly predicted a higher level of IA at Time 2. These results indicate that the associations between IA and depression and delinquency were bidirectional, echoing previous findings which suggested that pathological use of Internet can be caused by emotional and behavioral maladjustment in adolescence (Cerruti et al., 2017; Bağatarhan and Siyez, 2020) and also further worsen these problems (Yu and Shek, 2018; Chi et al., 2019). While most previous studies focused on one of the two predictions, especially the association between IA and depression (Lam, 2014), our study investigated the two aspects simultaneously and included delinquency as well, thus providing additional and comprehensive evidence for the dynamic relationship between IA and other developmental issues.

In line with previous findings, our results suggest that some adolescents are more vulnerable to the development of IA than others, including those having other emotional and behavioral symptoms (Cerniglia et al., 2017; Chung et al., 2019). It has been found that adolescents with depression and/or delinquency would suffer from increased negative experiences and painful feelings in personal and social life, such as loneliness, hopelessness, poor academic performance, conflicts with parents, and parent–child relationships (Kofler et al., 2011; Cerniglia et al., 2017; Zhang et al., 2018). As a result, Internet surfing may serve as a dissociative strategy to cope with the distress and frustrations (Davis, 2001; Cerniglia et al., 2017), as adolescents can share feelings with others in a virtual world and get recognition and responses as well. This may allow the adolescents feel accompanied and satisfied, which they are not able to have in the real world due to insufficient effective social connections (Yao and Zhong, 2014). Thus, adolescents with developmental problems tend to spend more time on the Internet, using a seemingly adaptive “self-soothing” for resolution of problems they are facing and relief of associated negative feelings. Obviously, future studies should further explore whether coping mechanisms would mediate the impact of negative emotions on IA.

Despite the possibility that Internet may benefit depressed and delinquent adolescents to some extent (such as providing peer support and a sense of achievement), the cross-lagged effects from IA to depression and delinquency were also significant in the current study, with relatively larger coefficients than the reverse effects. These findings indicate that overuse of the Internet could worsen developmental problems even to a larger extent. This echoes the previous observations that IA among adolescents acts as a precursor of low well-being, poor self-esteem, and loneliness rather than an outcome (Yao and Zhong, 2014; Yu and Shek, 2018). One interpretation is that addictive behavior, including IA, is associated with significant structural changes in brain regions involving in cognitive control and reward processing (Jorgenson et al., 2016). Such changes further result in impairments in adolescents’ emotional and behavioral capacity, leading to ineffective coping and developmental problems (Fayazi and Hasani, 2017; Yu and Zhou, 2021). Meanwhile, excessive Internet usage inevitably reduces face-to-face interactions with parents and peers, which may impair relationships with peers and family members and reduce social support (Wang et al., 2018; Xin et al., 2018), all of which in turn damper adolescent healthy development but lead to maladjustment in multiple aspects (Wang et al., 2018; Shek and Zhu, 2019; Zhu and Shek, 2021a).

While the overall bidirectional relationships between IA and depression and delinquency are supported in the present study, some previous findings failed to identify cross-lagged effects between IA and depression (Yao and Zhong, 2014) or only supported one direction causality (Yu and Shek, 2018). We argued that such inconsistent findings may partially be attributed to gender differences that were not examined in the previous studies. The present study found that boys reported higher levels of delinquency and IA than did girls, while gender was not significantly related to depression level. Besides, baseline depression significantly predicted subsequent IA in 1year only among boys but not among girls, which is in line with Liang et al.’s (2016) observation. These findings suggest that the relationship between adolescent IA and depression is dependent on gender. In particular, male adolescents are more prone to turn to Internet (e.g., playing online games) to alter or escape negative feelings associated with depression. It is possible that compared with girls, boys are less inclined to seek social support and use effective emotional regulation strategies and they are more likely to suppress or avoid emotional expression (Gross and John, 2003; Nolen-Hoeksema, 2012). This is particularly the case in Chinese contexts where boys are socialized to be tough and emotionally suppressive while girls are encouraged to be more sensitive (Yeh et al., 2017).

The reciprocal relationship between IA and delinquency did not vary across gender, although the effect of IA on delinquency appeared to be stronger among boys. These observations are not consistent with Liang et al.’s (2016) study, which found that IA significantly predicted future depression only among girls. The authors argued that compared with boys, girls with IA are more likely to reduce off-line social contact and suffer from social isolation, which often leads to depression. However, in our study, boys were more likely to show negative consequences of IA, especially behavioral problems. There are several explanations for this observation. First, it may be due to relatively higher levels of IA among boys. Second, it might be related to different ways in which the Internet is used by boys and girls. Previous studies found that compared with girls who usually use Internet to search and exchange information, boys use Internet more for pleasure through multiple online activities such as playing online games (Jones et al., 2009; Liang et al., 2016). It can be argued that the unique behavioral pattern underlying IA among boys may be more closely related to poor self-regulation and cognitive impulsivity, which eventually elevates the likelihood of delinquency. Nevertheless, the exact mechanisms underlying the present gender differences warrant further investigation in the future.

The present findings suggest that scholars, educators, youth workers, and parents need to pay attention to adolescents’ emotional and behavioral issues when designing prevention and intervention programs to deal with excessive use of Internet. At the same time, regulating adolescent children’s online activities may be a way to help prevent depression and delinquency. However, it is noteworthy that the values of regression coefficients between IA and delinquency and depression were not large, which suggests that IA can explain only a small proportion of variance in depression and delinquency and vice versa. Similar observations have been reported in previous studies (Liang et al., 2016; Tian et al., 2021). It is noted that the cross-lagged model is a conservative analytic tool that controls the effects of autoregression (i.e., the stability of key constructs) and covariates (age, gender, and family intactness in this study). Thus, the lagged path coefficients in cross-lagged models are normally not high (Adachi and Willoughby, 2015; Wu et al., 2020; Zhou et al., 2020). Researchers recommended that the so-called “small” effect in the longitudinal study should not be treated as “trivial” but actually “meaningful” (Adachi and Willoughby, 2015, p. 116).

Nevertheless, it can be argued that IA, depression, and delinquency are complex developmental disorders shaped by multiple ecological factors and causal pathways. For example, these developmental problems may be affected by individual (e.g., positive youth development attributes) and contextual factors (e.g., parenting and family functioning) simultaneously (Shek et al., 2018b; Zhou et al., 2020; Zhu and Shek, 2021a,b; Dou and Shek, 2021), which may lead to the associations among IA, depression, and delinquency. In this case, effective prevention and intervention youth programs may need to focus more on promoting adolescents’ competence and skills in a holistic manner instead of only targeting at eliminating one specific disorder, including IA (Shek et al., 2018a, 2020; Chung et al., 2019; Shek, 2019). Evidence in positive youth development research field strongly suggests that strengthening protective factors on both intrapersonal (e.g., emotional competence) and interpersonal (e.g., social skills) levels is a promising way to prevent and curb adolescent developmental problems, including depression, delinquency, and IA (Ciocanel et al., 2017; Taylor et al., 2017; Zhu and Shek, 2020a).

Although the reciprocal associations between IA and depression and delinquency and the gender differences elucidated in the present study have valuable theoretical and practical implications, the findings should be interpreted in light of several limitations. First, only two waves of data were collected for each variable, which makes it impossible to test whether the reciprocal associations would change over time. Obviously, there is a need to collect more waves of data over a longer time span in the future. Second, with two waves of data, we employed traditional CLPM, which could have poor performance when there is correlated trait variance in the constructs under investigation (Hamaker et al., 2015). Thus, random-intercepts CLPM (RI-CLPM) is recommended to partial out any between-person differences and ensure that the cross-lagged effects reliably reflect within-person fluctuations (Mulder and Hamaker, 2021). Therefore, in future studies, RI-CLPM should be used with at least three waves of data to further clarify the reciprocal relationships between IA and other emotional and behavioral problems. Third, this study did not examine why there are gender differences in the associations between IA and other developmental issues. Future research will benefit from including additional variables that may help explain the identified gender effect. Fourth, given that the participants in this study were recruited from four cities located in East, South, and Central China, the current findings may not be generalized to all Chinese adolescents. Future research could further involve Chinese adolescents living in other regions, such as North and West China, Hong Kong, and Macau. Fifth, because of administrative convenience for each participating school, we collected data at the beginning of a new semester, when adolescents may face increasing adaptation challenges and thus might report high levels of emotional and behavioral maladjustment. Although no such adverse effect was reported by the schools, future research could consider collecting data at a different time point (e.g., at the end of a semester).

## Data Availability Statement

The raw data supporting the conclusions of this article will be made available by the authors, without undue reservation.

## Ethics Statement

The studies involving human participants were reviewed and approved by Human Subjects Ethics Subcommittee at The Hong Kong Polytechnic University. Written informed consent to participate in this study was provided by the participants’ legal guardian/next of kin.

## Author Contributions

XZ contributed to the design of the project, data collection, and data interpretation of the work, drafted the work, and revised it based on the critical comments provided by DS. DS conceived the project, obtained the funding, and edited the manuscript. CC drafted the work. All authors contributed to the article and approved the submitted version.

## Funding

This paper and the two-wave longitudinal study in the Tin Ka Ping P.A.T.H.S. Project are financially supported by Tin Ka Ping Foundation.

## Conflict of Interest

The authors declare that the research was conducted in the absence of any commercial or financial relationships that could be construed as a potential conflict of interest.

## Publisher’s Note

All claims expressed in this article are solely those of the authors and do not necessarily represent those of their affiliated organizations, or those of the publisher, the editors and the reviewers. Any product that may be evaluated in this article, or claim that may be made by its manufacturer, is not guaranteed or endorsed by the publisher.
